# Program in BASIC to combine data from two different selective detectors and its application for screening of pesticides in residue analysis

**DOI:** 10.1155/S1463924684000043

**Published:** 1984

**Authors:** H.-J. Stan, H. Goebel

**Affiliations:** Institut für Lebensmittelchemie der Technischen Universität Berlin Müller-Breslau-Straβle Berlin 12 1000 Germany


Journal of Automatic Chemistry, Volume 6, Number 1 (January–March 1984), pages 14–20

### Program in BASIC to combine data from two different selective detectors and its application for screening of pesticides in residue analysis

**H.-J. Stan and H. Goebel**

*Institut für Lebensmittelchemie der Technischen Universität Berlin, Müller-Breslau-Straße, 1000 Berlin 12, FR Germany*

### Introduction

For the analysis of pesticide residues in food, gas chromatography with selective detectors is established internationally as the most suitable method. The application of electron capture (ECD) and nitrogen-phosphorus (NPD) detectors enables the selective detection of contaminants at trace level in the presence of a multitude of compounds extracted from the matrix, which do not respond to these detectors.

The number of compounds used in agriculture for plant protection and the variety of pollutants in the environment has increased to such a level that it is impossible to separate them all in a single chromatogram, despite using high-performance capillary columns. These capillary columns allow the retention times of compounds to be determined with a very high accuracy and good reproducibility. The high resolution facilitates the differentiation of substances belonging to the same structural class as organophosphate pesticides (PP) or chlorinated pesticides (CP).

When splitting the effluent of the capillary column to both selective detectors, additional information about the identity of the individual compounds can be obtained from the chromatograms by calculating the response ratios.

A concept has recently been developed for automated pesticide residue analysis—realizing it by means of a gas chromatograph with options for BASIC programming, dual-channel operation and automatic liquid sampling [1 and 2].

The BASIC program, which controls the automated analysis, calculates the residue concentrations and evaluates the degree of certainty of the pesticides found is presented here.

### Materials and methods

The pesticide residue analysis was performed with a gas chromatograph (HP 5880 A, Hewlett Packard, Palo Alto, California, USA) using capillary columns (BP 1, SGE-Scientific Glass Engineering, Australia) and effluent splitting to the two selective detectors (NPD and ECD). The signals from detectors are processed in a dual-channel integrator connected to two terminals, allowing both chromatograms to be recorded in parallel. The manufacturer's operating system and chromatography programs are used for data collection, immediate recording of the chromatograms, recognition of calibrated peaks and quantitation. The internal standard method is generally used. The instrument is also equipped with an autosampler, HP 7671 A, and the microprocessor's memory is extended by a cartridge tape unit.

The procedure of the complete pesticide analysis has already been described [1 and 2].

### The program

The HP 5880 A was launched as the first gas chromatograph which could be programmed in BASIC; this allows it to execute individual calculations, to format reports and to control the autosampler. The authors' program was developed to fulfil the following requirements:

(1) The ability to run a food sample with parallel signal processing in two channels and storage of the data.
(2) Calculation of peaks found by means of several calibration tables.
(3) Evaluation of the results by comparing the peaks identified in the two channels.
(4) Summarizing final results in a clearly arranged report.

The HP 5880 A was designed to store one calibration table in each channel. However, the large number of pesticides included in the authors' analytical method requires at least three calibration mixtures with the three corresponding calibration tables in each channel. Therefore the calibration tables have to be saved on an external memory: in this case a tape. The calibration tables are included in the three 'Analysis files'. Each file contains two calibration tables generated by dual-channel recording of one calibration mixture. Additionally, the analysis files contain the parameter settings of the instrument to run the gas chromatographic analysis. A special problem arises from the fact that communication between the two channels is limited—calculations can easily be performed, but processing in one channel cannot be controlled by the other one. Therefore two separate programs have been created, these are synchronized by means of waiting loops.

The programs are documented in figures 1 and 2; the REM statements should make them almost self-explanatory.

For readers unfamiliar with the HP 5880 A, a list of some of the commands for activating special procedures and functions integrated in the program might be helpful:

START AUTO SEQ X, X: Starts the automatic sampler. The two numbers define the first and last bottle.

RECALIB: The areas from the calibration mixtures found in the most recent run and the amounts from the original calibration run of the same mixture are used to calculate new response factors.

RECALIB RUN TIME: The retention times in the calibration table are replaced by real times found in the most recent run.

SAMPLE $: Returns the number of the sampler bottle in the tray.

ID $: Returns the sample names from the sample table.

DETECTOR A O: NPD is switched off.
AMT (I): Returns the concentration of a specified peak calibrated.
HEAD $: Returns the title from the calibration table.

### Application to a real food sample

The automated pesticide residue analysis controlled by the BASIC program described has been designed as a screening procedure. As already mentioned, a complete residue analysis in food of unknown origin cannot be performed on a single capillary column: so the aim of automated screening is to provide the analyst with information about contaminants that may be present in the specified sample. All of the suspected pesticides listed in the final report are analysed using an independent method of confirmation [1 and 2]. Examples of final reports from the two channels are shown in figures 3 and 4. All peaks identified by means of the data stored for each calibration mixture are printed, and those responding to both detectors are compared by applying their specific response ratios. The deviation from the value calculated from the calibration data is reported as a percentage. A difference of more than 30% is usually an indication that the peak found does not correspond to the substance calibrated. Also, all compounds identified with only one detector are indicated, as well as those belonging to ‘critical pairs’ in chromatography and those responding to both detectors.

```

LIST PRGM

PROGRAM:            (ANNOTATION OFF)

 10  PRINT “LEADING PROGRAM (CHANNEL 1) FOR RESIDUE ANALYSIS”

 20  PRINT “PARALLEL IN NPD AND ECD”

 30  REM

 40  REM ********** NPD (SIGNAL A): CHANNEL 1

 50  REM ********** ECD (SIGNAL D): CHANNEL 2

 60  REM ********** COLUMN: METHYLSILICONE- BP1

 70  REM ********** INTERNAL STANDARDS FOR NPD: NT (PT)

 80  REM **********                   FOR ECD: ALDRIN (1,2,3- TCB)

 90  REM ********** CALIBRATION TABLES AND INSTRUMENT SETPOINTS

100  REM ********** IN ANALYSIS FILES 1,2 AND 3

110  PRINT

120  PRINT “PUSH Y IF LOADING THE THREE STANDARD MIXTURES INTO THE TRAY”

130  INPUT “AS FOLLOWS: PPI, PPII, CPI AND STARTING CHANNEL 2”, A$

140  IF A$< >“Y” THEN 120

150  PRINT

160  PRINT “CREATION OF SAMPLE TABLE”

170  PRINT

180  PRINT “START WITH 4 AND CONCLUDE BY PUSHING EXIT”

190  PRINT

200  SAMPLE TBL

210  INPUT “HOW MANY BOTTLES ARE IN THE TRAY?”,N

220  LET C=N-3

230  GET ANALYSIS 1 DEVICE# 6

240  OVEN TEMP ANNOTATION OFF

250  LIST CLOCK TIME

260  START AUTO SEQ 1,1

270  RECALIB

280  RECALIB RUN TIME

290  WAIT 0.2

300  DELETE ANALYSIS 1 DEVICE# 6

310  SAVE ANALYSIS 1 DEVICE# 6

320  GOSUB 2030

330  GET ANALYSIS 2 DEVICE# 6

340  OVEN TEMP ANNOTATION OFF

350  START AUTO SEQ 2,2

360  RECALIB

370  RECALIB RUN TIME

380  WAIT 0.2

390  DELETE ANALYSIS 2 DEVICE# 6

400  SAVE ANALYSIS 2 DEVICE# 6

410  GOSUB 2030

420  GET ANALYSIS 3 DEVICE# 6

430  OVEN TEMP ANNOTATION OFF

440  START AUTO SEQ 3,3

450  RECALIB

460  RECALIB RUN TIME

470  WAIT 0.2

480  DELETE ANALYSIS 3 DEVICE# 6

490  SAVE ANALYSIS 3 DEVICE# 6

500  GOSUB 2030

510  WAIT 1

520  GET ANALYSIS 1 DEVICE# 6

530  REM

540  REM ********** INJECTION OF C SAMPLES AND STORING THE DATA ON TAPE

550  REM

560  FOR N1=4 TO N

570  OVEN TEMP ANNOTATION OFF

580  ATTN 2↑2

590  THRESHOLD 1

600  START AUTO SEQ N1,N1

```

```

610  PRINT SAMPLE$,ID$

620  LIST SIGNAL

630  WAIT 0.5

640  EXECUTE E, "SAVE REPORT "&VAL$(N1)&" DEVICE# 6"

650  NEXT N1

660  PRINT

670  DETECTOR A 0

680  PRINT

690  PRINT TAB(15); "REGISTER OF PESTICIDE RESIDUES OF"; C; "SAMPLES"

700  PRINT

710  PRINT

720  IMAGE X,2A,4X,12A,4X,12A,4X,DD.DD,4X,DD.DD,4X,DD.DD,4X,DDDDDD

730  IMAGE X,2A,4X,12A,4X,12A,4X,5A,4X,5A,4X,6A,4X,6A

740  PRINT USING 730; "NO", "SAMPLE", "NAME", "PPM", "RT", "EXP.RT", "AREA"

750  PRINT

760  PRINT

770  FOR N1=4 TO N

780  PRINT "CALCULATED FROM STANDARD MIXTURE I FOR P-COMPOUNDS"

790  PRINT

800  WAIT 0.5

810  REM

820  REM ********** LOADING THE REPORTS INTO MEMORY AND COMPARISON WITH

830  REM ********** THREE STANDARD MIXTURES

840  REM

850  EXECUTE E, "GET REPORT "&VAL$(N1)&" DEVICE# 6"

860  GOSUB 1040

870  WAIT 1

880  GET ANALYSIS 2 DEVICE# 6

890  PRINT

900  PRINT "CALCULATED FROM STANDARD MIXTURE II FOR P-COMPOUNDS"

910  PRINT

920  GOSUB 1040

930  WAIT 1

940  GET ANALYSIS 3 DEVICE# 6

950  PRINT

960  PRINT "CALCULATED FROM STANDARD MIXTURE FOR N-COMPOUNDS"

970  PRINT

980  GOSUB 1040

990  GOTO 1320

1000 REM

1010 REM ********** PRINTING REPORTS AND COMPARISON OF RESULTS

1020 REM ********** BETWEEN ECD AND NPD

1030 REM

1040 FOR I=1 TO #PEAKS

1050 IF AMT(I)> =0.01 THEN 1070

1060 GOTO 1080

1070 PRINT USING 720; SAMPLE$, ID$, NAME$(I),AMT(I),RT(I),EXPRT(I),AREA(I)

1080 NEXT I

1090 PRINT

1100 FOR I=1 TO #PEAKS

1110 LET U$=NAME$(I)

1120 LET F=AMT(I)

1130 FOR J=1 TO #PEAKS(2)

1140 IF AMT(I)<0.01 THEN 1290

1150 IF NAME$(J,2)=U$ THEN 1170

1160 GOTO 1280

1170 IF AMT(J,2)> =0.01 THEN 1190

1180 GOTO 1280

1190 LET E=AMT(J,2)

1200 IF F> =E THEN 1220

1210 IF E> =F THEN 1240

1220 LET Y=(F-E)*100/F

1230 GOTO 1250

1240 LET Y=(E-F)*100/E

1250 IMAGE "THE DIFFERENCE BETWEEN THE DETECTORS AMOUNTS TO ",DDD,"%"

1260 PRINT USING 1250;Y

1270 PRINT "FOR",NAME$(J,2)

1280 NEXT J

1290 NEXT I

1300 PRINT

1310 RETURN

1320 IMAGE 72("#")

1330 PRINT USING 1320

1340 PRINT

1350 GOSUB 1580

1355 WAIT 1

1360 GET ANALYSIS 2 DEVICE# 6

1370 GOSUB 1680

1380 GET ANALYSIS 1 DEVICE# 6

1390 GOSUB 1680

1400 RRINT

1410 PRINT USING 1320

```

```

1420 PRINT

1430 NEXT N1

1440 REM

1450 REM ********** FOOT NOTES TO THE FINAL REPORT

1460 REM

1470 PRINT TAB(10); "* BELONGS TO A CRITICAL PESTICIDE THAT INDICATES"

1480 PRINT TAB(13); "SIMILAR RETENTION TIME TO A COMPOUND OF ANOTHER"

1490 PRINT TAB (27); "STANDARD MIXTURE"

1500 PRINT

1510 PRINT TAB(10); "+BELONGS TO A COMPOUND RESPONDING TO ECD AND NPD"

1520 PRINT

1530 GET REPORT 4 DEVICE# 6

1540 STOP

1550 REM

1560 REM ********** FINAL EVALUATIONS

1570 REM

1580 FOR J=1 TO #PEAKS(2)

1590 LET Y$=NAME$(J,2)

1600 FOR I=1 TO #PEAKS

1610 IF NAME$(I)=Y$ THEN 1660

1620 IF NAME$(J,2)="ALDRIN" THEN 1660

1630 IF AMT (J,2)<0.01 THEN 1660

1640 NEXT I

1650 PRINT NAME$(J,2),"IS IDENTIFIED WITH ECD IN";ID$

1660 NEXT J

1670 GOTO 1780

1680 FOR I=1 TO #PEAKS

1690 LET X$=NAME$(I)

1700 FOR J=1 TO #PEAKS(2)

1710 IF NAME$(J,2)=X$ THEN 1770

1720 IF NAME$(I)="PT" THEN 1770

1730 IF NAME$(I)="NT" THEN 1770

1740 IF AMT(I)<0.01 THEN 1770

1750 NEXT J

1760 PRINT NAME$(I), "IS IDENTIFIED WITH NPD IN";ID$

1770 NEXT I

1780 FOR I=1 TO #PEAKS

1790 LET U$=NAME$(I)

1800 LET F=AMT(I)

1810 FOR J=1 TO #PEAKS(2)

1820 IF NAME$(J,2)=U$ THEN 1840

1830 GOTO 1960

1840 IF AMT(J,2)> =0.01 THEN 1860

1850 GOTO 1960

1860 LET E=AMT(J,2)

1870 IF F> =E THEN 1890

1880 IF E> =F THEN 1910

1890 LET Y=(F-E)*100/F

1900 GOTO 1920

1910 LET Y=(E-F)*100/E

1920 IF Y< =30 THEN 1950

1930 GOTO 1970

1940 PRINT

1950 PRINT NAME$(J,2), "IS SUSPECTED IN";ID$

1960 NEXT J

1970 NEXT I

1980 RETURN

1990 REM

2000 REM ********** PRINTING CALIBRATION REPORTS OF STANDARD MIXTURES

2010 REM ********** INCLUDING RELATIVE RETENTION TIMES

2020 REM

2030 FOR I=1 TO #PEAKS

2040 IF NAME$(I)="NT" THEN 2060

2050 GOTO 2070

2060 LET A=RT(I)

2070 NEXT I

2080 PRINT HEAD$

2090 PRINT

2100 PRINT "CAL", "NAME", "PPM", "RT", "REL.RT"

2110 PRINT

2120 FOR I=1 TO #PEAKS

2130 PRINT CAL#(I),NAME$(I),AMT(I),RT(I),RT(I)/A

2140 NEXT I

2150 RETURN

```

*Figure 1. The program for the NPD channel (leading program).*

```

LIST PRGM

PROGRAM:            (ANNOTATION OFF)

  10  PRINT "PROGRAM (CHANNEL 2) FOR RESIDUE ANALYSIS"

  20  PRINT "PARALLEL IN ECD AND NPD"

  30  REM

  40  REM ********** NPD (SIGNAL A): CHANNEL 1

  50  REM ********** ECD (SIGNAL D): CHANNEL 2

  60  PRINT

  70  INPUT "HOW MANY BOTTLES ARE IN THE TRAY?",N

  80  START

  90  RECALIB

 100  RECALIB RUN TIME

 110  GOSUB 1070

 120  START

 130  RECALIB

 140  RECALIB RUN TIME

 150  GOSUB 1070

 160  START

 170  RECALIB

 180  RECALIB RUN TIME

 190  GOSUB 1070

 200  REM

 210  REM ********** INJECTION OF SAMPLES AND STORING THE DATA ON TAPE

 220  REM

 230  FOR B1=104 TO N+100

 240  ATTN 2↑9

 250  THRESHOLD 9

 260  START

 270  LIST CLOCK TIME

 280  PRINT SAMPLE$,ID$

 290  PRINT "SIGNAL D"

 300  EXECUTE E, "SAVE REPORT "&VAL$(B1)&" DEVICE# 16"

 310  NEXT B1

 320  PRINT

 330  PRINT

 340  PRINT TAB(15); "REGISTER OF PESTICIDE RESIDUE OF"; N-3; "SAMPLES"

 350  PRINT

 360  PRINT

 370  IMAGE X,2A,4X,12A,4X,12A,4X,DD.DD,4X,DD.DD,4X,DD.DD,4X,DDDDDD

 380  IMAGE X,2A,4X,12A,4X,12A,4X,5A,4X,5A,4X,6A,4X,6A

 390  PRINT USING 380; "NO", "SAMPLE", "NAME", "PPM", "RT", "EXP.RT", "AREA"

 400  PRINT

 410  PRINT

 420  FOR B1=104 TO N+100

 430  PRINT

 440  PRINT

 450  PRINT "CALCULATED FROM STANDARD MIXTURE I FOR P-COMPOUNDS"

 460  REM

 470  REM ********** LOADING THE REPORTS INTO MEMORY AND COMPARISON WITH

 480  REM ********** THREE STANDARD MIXTURES

 490  REM

 500  EXECUTE E, "GET REPORT "&VAL$(B1)&" DEVICE# 16"

 510  REM

 520  REM ********** WAITING LOOP FOR SYNCHRONISATION

 530  REM ********** WITH THE MAIN CHANNEL 1

 540  REM

 550  LET W$=SAMPLE$(2)

 560  IF W$=SAMPLE$(1) THEN 590

 570  WAIT 0.2

 580  GOTO 550

 590  GOSUB 870

 600  REM

 610  REM ********** WAITING LOOP FOR SYNCHRONISATION

 620  REM ********** WITH THE MAIN CHANNEL 1

 630  REM

 640  LET A$=HEAD$

 650  IF A$="STANDARD MIXTURE II IN ECD" THEN 680

 660  WAIT 0.2

 670  GOTO 640

 680  PRINT

 690  PRINT "CALCULATED FROM STANDARD MIXTURE II FOR P-COMPOUNDS"

 700  GOSUB 870

 710  REM

 720  REM ********** WAITING LOOP FOR SYNCHRONISATION

 730  REM ********** WITH THE MAIN CHANNEL 1

 740  REM

 750  LET B$=HEAD$

 760  IF B$="STANDARD MIXTURE FOR CHLORINATED COMPOUNDS IN ECD" THEN 790

 770  WAIT 0.2

 780  GOTO 750

 790  PRINT

```

```

800  PRINT "CALCULATED FROM STANDARD MIXTURE FOR CHLORINATED COMPOUNDS"

810  GOSUB 870

820  GOTO 930

830  REM

840  REM ********** PRINTING REPORTS AND COMPARISON OF RESULTS

850  REM ********** BETWEEN ECD AND NPD

860  REM

870  FOR I=1 TO #PEAKS

880  IF AMT(I)> =0.01 THEN 900

890  GOTO 910

900  PRINT USING 370; SAMPLE$, ID$, NAME$(I), AMT(I), RT(I), EXPRT(I), AREA(I)

910  NEXT I

920  RETURN

930  WAIT 0.2

940  REM

950  REM ********** WAITING LOOP FOR SYNCHRONISATION

969  REM ********** WITH THE MAIN CHANNEL 1

970  REM

980  LET V$=SAMPLE$(2)

990  IF V$< >SAMPLE$(1) THEN 1010

1000 GOTO 930

1010 NEXT B1

1020 STOP

1030 REM

1040 REM ********** PRINTING CALIBRATION REPORTS OF THE STANDARD MIXTURES

1050 REM ********** INCLUDING RELATIVE RETENTION TIMES

1060 REM

1070 FOR I=1 TO #PEAKS

1080 IF NAME$(I)="ALDRIN" THEN 1100

1090 GOTO 1110

1100 LET A=RT(I)

1110 NEXT I

1120 PRINT HEAD$

1130 PRINT

1140 PRINT "CAL", "NAME", "PPM", "RT", "REL.RT"

1150 PRINT

1160 FOR I=1 TO #PEAKS

1170 PRINT CAL#(I),NAME$(I),AMT(I),RT(I),RT(I)/A

1180 NEXT I

1190 RETURN

```

*Figure 2. The program for the ECD channel.*

REGISTER OF PESTICIDE RESIDUE OF 1 SAMPLES

| NO | SAMPLE | NAME | PPM | RT | EXP.RT | AREA |
|---|---|---|---|---|---|---|
| CALCULATED FROM STANDARD MIXTURE I FOR P-COMPOUNDS | | | | | | |
| 4 | PEACHES | PHOSPHAMI+ | .54 | 13.97 | 13.86 | 1763 |
| 4 | PEACHES | PARATH-ME+* | 1.48 | 14.21 | 14.20 | 49932 |
| 4 | PEACHES | MALATHION+ | 2.69 | 16.49 | 16.57 | 16716 |
| 4 | PEACHES | ALDRIN | 2.00 | 16.85 | 16.85 | 100997 |
| 4 | PEACHES | PARATHION+* | .20 | 17.21 | 17.14 | 2577 |
| CALCULATED FROM STANDARD MIXTURE II FOR P-COMPOUNDS | | | | | | |
| 4 | PEACHES | DICHLOFENT+* | 3.37 | 14.21 | 14.18 | 49932 |
| 4 | PEACHES | PARAOXON+* | .59 | 14.98 | 15.05 | 5607 |
| 4 | PEACHES | ALDRIN | 2.00 | 16.85 | 16.85 | 100997 |
| 4 | PEACHES | DURSBAN+* | .10 | 17.21 | 17.18 | 2577 |
| CALCULATED FROM STANDARD MIXTURE FOR CHLORINATED COMPOUNDS | | | | | | |
| 4 | PEACHES | CHLORFPROP-M | .79 | 7.67 | 7.60 | 2719 |
| 4 | PEACHES | HEPTACHLOR* | .30 | 14.98 | 14.97 | 5607 |
| 4 | PEACHES | ALDRIN | 2.00 | 16.85 | 16.85 | 100997 |

*Figure 3. Print-out from the ECD channel for a screening run of pesticides in peaches.*

REGISTER OF PESTICIDE RESIDUES OF 1 SAMPLES

| NO | SAMPLE | NAME | PPM | RT | EXP.RT | AREA |
|---|---|---|---|---|---|---|
| CALCULATED FROM STANDARD MIXTURE I FOR P–COMPOUNDS | | | | | | |
| 4 | PEACHES | PARATH–ME+* | 1.60 | 14.20 | 14.32 | 1088 |
| 4 | PEACHES | PARATHION+ * | .10 | 17.20 | 17.28 | 27 |
| 4 | PEACHES | NT | 2.00 | 18.19 | 18.19 | 1055 |

THE DIFFERENCE BETWEEN THE DETECTORS AMOUNTS TO 8%
FOR PARATH-ME+*
THE DIFFERENCE BETWEEN THE DETECTORS AMOUNTS TO 52%
FOR PARATHION+*

| NO | SAMPLE | NAME | PPM | RT | EXP.RT | AREA |
|---|---|---|---|---|---|---|
| CALCULATED FROM STANDARD MIXTURE II FOR P–COMPOUNDS | | | | | | |
| 4 | PEACHES | DICHLOFENT+* | 5.11 | 14.20 | 14.28 | 1088 |
| 4 | PEACHES | DURSBAN+* | .07 | 17.20 | 17.31 | 27 |
| 4 | PEACHES | NT | 2.00 | 18.19 | 18.19 | 1055 |

THE DIFFERENCE BETWEEN THE DETECTORS AMOUNTS TO 34%
FOR DICHLOFENT+*
THE DIFFERENCE BETWEEN THE DETECTORS AMOUNTS TO 35%
FOR DURSBAN+*

| NO | SAMPLE | NAME | PPM | RT | EXP.RT | AREA |
|---|---|---|---|---|---|---|
| CALCULATED FROM STANDARD MIXTURE FOR N–COMPOUNDS | | | | | | |
| 4 | PEACHES | NT | 2.00 | 18.19 | 18.19 | 1055 |

################################################################################

CHLORFPROP–M IS IDENTIFIED WITH ECD IN PEACHES
HEPTACHLOR* IS IDENTIFIED WITH ECD IN PEACHES
PARATH–ME+* IS SUSPECTED IN PEACHES

################################################################################

* BELONGS TO A CRITICAL PESTICIDE THAT INDICATES SIMILAR RETENTION TIME TO A COMPOUND OF ANOTHER STANDARD MIXTURE
\+ BELONGS TO A COMPOUND RESPONDING TO ECD AND NPD

*Figure 4. Print-out from the NPD channel for the same screening run as in figure 3, including final report.*

### Discussion

Figures 3 and 4 show the parallel print-outs from the two terminals reporting a screening run for pesticide residues in peaches. By means of this example, the utility and the limits of our program for evaluating food samples are briefly discussed.

After the clean-up, a number of substances responding to the ECD are generally found in a chromatogram and several are recognized as calibrated pesticides in the screening run. In figure 3 a total of nine pesticides are indicated, together with the internal standard (aldrin). Only these nine compounds out of about 80 pesticides incorporated in three calibration mixtures may be present in the sample. Processing of the NPD signals recorded simultaneously in the other channel results in four organophosphorous pesticides and no nitrogen-containing pesticide (figure 4). For all of the compounds responding to both detectors a calculation of the response ratios and comparison with their calibrated values leads to the discrimination of 'parathion', dichlofenthion' and 'dursban', whereas the response ratio of 'parathion methyl' is close to the calibrated one. Therefore parathion methyl is announced in the final report as suspected. This screening run permits no further decision about 'chlorfenprop methyl' and 'heptachlor'.

All pesticides marked with a cross in figure 3 are organophosphorous compounds and so they also have to respond to the NPD. As there are no corresponding signals for 'phosphamidon', 'malathion' and 'paraoxon' in the NPD report of this example, all three substances are eliminated and do not appear in the final report. This discrimination procedure results in the final proposal of chlorfenprop methyl, heptachlor and parathion methyl as possibly being present in this sample.

The program proved to be a great help in routine analysis for selecting the positive food samples after screening. A major drawback is the time-consuming data transfer between the tape and the gas chromatograph's memory.

The analyst has to inspect the chromatograms of both detectors in order to evaluate the quality of the separation and the performance of the chromatographic system. After this, the computer supports him by handling the huge amount of information produced by a series of screening runs.

### References

1. GOEBEL, H. and STAN, H.-J. in J. Rijks (ed.), *Proceedings of the Fifth International Symposium on Capillary Chromatography* (Elsevier, Amsterdam, 1983), 557.
2. STAN, H.-J. and GOEBEL, H., *Journal of Chromatography* (in press).

